# Molecular epidemiology of antiretroviral resistance in therapy-experienced HIV-1 patients in Cuba (2009)

**DOI:** 10.1186/1758-2652-13-S4-P141

**Published:** 2010-11-08

**Authors:** L Pérez, J Aleman, C Correa, J Pérez, C Fonseca, C Aragones, D Pérez, A Alvarez, AM Vandamme, V Kourí, K Van Laethem

**Affiliations:** 1 Institute of Tropical Medicine, Virology, Havana City, Cuba; 2 Institute of Tropical Medicine, Hospital, Havana City, Cuba; 3 Institute of Tropical Medicine, Havana City, Cuba; 4 Rega Institute for Medical Research, Katholieke Universiteit Leuven, Leuven, Belgium

## Background

In 2001, Cuba launched a national ARV access program to provide ARV therapy free of cost. The first-line therapy combinations are composed of two nucleoside reverse transcriptase (RT) inhibitors (NRTIs) (AZT+3TC or d4T+3TC) associated with a non-nucleoside RT inhibitor (NNRTI) (NVP or EFV) or a protease inhibitor (PI) (IDV).

## Purpose of the study

To identify resistance mutations in HIV-1 isolated obtained from Cuban treated patients presenting clinical and/or immunological failure.

## Methods

Plasma samples from 84 HIV-1 infected patients were collected from June to November 2009 at the Tropical Medicine Institute from Havana City. Viral RNA fragments corresponding to the PR and RT region were amplified, sequenced and sub typed. Drug resistance was interpreted according to http//hivdb.stanford.edu.

## Results

The most frequently found subtypes and recombinants were: B (40.4%), D (32.1%, presumably CRF19_cpx), C (9.5%) and CRF18_cpx (8.3%). The PI mutations, I54VML, M46IL and L90MVL were found in 61.7, 57.4 and 53.1% of cases, respectively. Whereas for RTI, primary mutations at positions M184V, T215Y, D67N, Y181C and K103N were present in 98.6, 67.1, 43.8, 47.5 and 40.9% of subjects, respectively. A high level resistance to antiretroviral drug was observed (NRTI=86.9%, NNRTI=72.6% and PI=55.9%). Full class resistance (NRTI/NRTI/PI) was found in 22 patients (26.1%), who presented history of several changes of treatment (media of 1.35 treatment changes per year, from 4 to 13 year under treatment).

## Conclusions

The present study reveals a high rate of resistance compared with other international reports suggesting that alternative strategies for initial therapy and lab monitoring should be considered. These findings also warrant the study of antiviral resistance in therapy-naïve patients.

